# Notes to Serve as the Basis of an Essay on the Effects of Dislocations, Sprains, and Fractures, into or near the Elbow-Joint

**Published:** 1856-04

**Authors:** J. W. Freer

**Affiliations:** Professor of Anatomy in Rush Medical College


ARTICLE II.—*Notes to serve as the basis of an Essay on the Effects of Dislocations, Sprains, and Fractures into or near the Elbow-Joint.* By J. W. FREER, M.D., Professor of Anatomy in Rush Medical College.

There is no fracture or dislocation more difficult to diagnosticate with certainty, or to treat with success, than those of the elbow; none which more frequently leave behind, no matter with what skill and care the treatment may have been conducted, more or less of deformity and imperfection in the movement and strength of the member.

These defects we do not propose to consider in general, but only in so far as they are peculiar to the injuries of the elbow-joint, as they seem to have been passed lightly over by surgical writers.

Sir Chas. Bell, speaking of fractures of the humerus, which extend into the elbow-joint, says, "Begin early to move the joint, or you will have anchylosis," and he adds, "this is an unlucky case for a young practitioner. The inflammation runs high, and stiffness of the joint and deformity is likely to be the result."—*Institutes of Surgery*.

Sir Astley Cooper says, in reference to fractures of the elbow-joint, that passive motion should be used to prevent the occurrence of anchylosis, and adds, "but even after the most careful and judicious means that can be adopted, there is sometimes considerable loss of motion; and when the accident has not been understood, or carefully treated, the deformity and loss of motion becomes very considerable."—*Dislocations and Fractures of the Joints*.

Samuel Cooper describes stiffness of the elbow-joint as a frequent result not only of fractures, but also of sprains and dislocations.—*Surgical Dictionary.*

Dessault quotes Petit, Heister, and Duvernay, to show the extending of a fracture into a joint, is an extremely dangerous complication, and says that anchylosis was regarded by them as the most favorable termination which could be expected.—*Surgical Works.*

Chelius, on the subject of fractures through the condyles of the humerus, says the inflammation is always very considerable, and often the motions are interfered with, or completely destroyed.

A recent writer says, in regard to fractures in the elbow-joint, "Under all the circumstances in which this form of accident occurs, it is right we should be prepared for the possible loss of the articulation."—*Skey's Operative Surgery.*

Gibson states, in regard to the same accident, "The injuries are often followed by high inflammation, anchylosis, and deformity of the whole arm."

Sanson, in his extremely elaborate work, says, "The prolonged repose of a joint, during the treatment of fractures, particularly if they are near the extremity of the bones, give to the surrounding parts such stiffness that the muscles, weakened by long inaction, cannot overcome it; and if this resistance is overcome by force, it produces a rough sound caused by the friction of the surfaces, accompanied by severe pain."—*New Elements of Medical and Surgical Pathology.*

Boyer, on the same subject, remarks, "Simple fractures always leave behind a stiffness, which sometimes is sufficiently considerable to merit the name of anchylosis. If the fracture is near a joint, the danger is increased; and if the member is kept in a dressing much longer than the usual time, the rigidity of ligaments and surrounding tissues may be such that it will be difficult, if not impossible, to re-establish the movements. Moreover, the articular surfaces, if left in this state, may adhere and form a complete anchylosis."—*Treatise on Surgical Diseases.*

The authors we have cited, express the opinions of surgical writers on the subject; and it is perhaps unnecessary to cite more, but we will add that of Malgaigne, whose work on the subject of fractures may be justly regarded as paramount authority on all points of which it treats. Speaking of fractures of the internal condyle of the humerus, he says, "The inflammation of the elbow draws after it, in the cure, almost inevitably a stiffness of the joint, which is very obstinate;" and again, "The prognosis is not serious in what concerns the fracture; but it must be known that stiffness of the joint is almost inevitable."

One essential point of the treatment, is to guard against anchylosis.—*Treatise on Fracture.*

It will be scarcely necessary to cite authorities to show that atrophy is a constant attendant on joints stiff from fracture, dislocations, and sprains. Physiology teaches us that whenever, from any cause, the muscles of a member are incapable of acting, atrophy is the inevitable result.

Marjolin and Hunter have, however, dwealt in so distinct a manner on the effect of injuries, that I will cite a few words from each. "It is not rare to see atrophy of a member result from a severe injury, and take place both above and below it." Marjolin attributes this atrophy to the injury of the nerves as well as to rest and inactivity of the muscles. It is remarkable that an injury done to tendons, ligaments, fasciæ, and especially of the sprain kind, impairs the muscles more than when the muscles themselves are injured, so that these muscles appear to sympathize with those parts of little action, and become wasted in consequence. The limb wastes and is œdematous."—*John Hunter's Works.*

In case of injury of the elbow, whether fracture of the internal condyle or dislocation, it rarely happens that the ulnar nerve entirely escapes injury, it is either bruised directly or pressed upon by the displacement, so that not unfrequently partial paralysis remains, particularly if suitable treatment is neglected.

Malgaigne says, "In these cases the ulnar nerve is wounded, either, as I think, by direct shock, or according to the opinion of Granger, by the pressure of the apophysis, upon it. Granger cites a remarkable example of this kind. It was a child eight years old, which fell upon the elbow.

The inflammation was violent, and the stiffness difficult to overcome. Nevertheless, three months after the accident, the child had recovered the full use of the joint, but the ulnar nerve remained paralyzed."—*Treatise on Fractures.*

Proper treatment, employed after several years, restored the sensibility and movements of the part. Granger has witnessed the same phenomena in two other cases.

Astley Cooper cites the following case. "I saw a girl, who, by falling upon the elbow, had fractured the olecranon, and also broke the internal condyle of the os humeri; the cubital nerve had also been injured, for the little finger and half the ring were benumbed."—*On Dislocation and Fracture of the Joints.*

The direct cause and nature of the stiffness of joints, resulting from fractures, dislocations, and sprains deserve a moment's notice. This, from whatever cause it may arise, and to whatever degree it may exist, is often called by the general term anchylosis, although there is no propriety in calling a simple rigidity, resulting from long continued repose, by the same term which is used to designate a consolidation of two bones into one. This distinction is kept in view by Samuel Cooper, who remarks, "After the cure of fractures, a certain stiffness generally remains in the adjacent joint, but this is different from anchylosis; but this is lost sight of by most writers, who, in the desire to systematize, have classed together states quite distinct from each other in their nature. Anchylosis, when used to embrace every variety of stiffness, is divided into true and false, and as the latter often results in the former, they are also styled complete and incomplete.

The false, incomplete, or spurious anchylosis may result from contraction of the tendons or ligaments, from effusion of lymph around or into the joint, from absence of the synovia, from long continued immobility, etc. The true anchylosis, as we have stated, consists in the consolidation of the bones into one.

Anchylosis very often occurs after fractures in the vicinity of the joints, after sprains and dislocations, after white swellings, etc. Malgaigne gives, in his work on fractures, a minute description of the successive stages through which the parts surrounding a joint pass after sprains, dislocations, fractures, or other injuries, to the formation of a perfect anchylosis. First, effusion of blood, effusion of lymph, which becomes organized; shortening of the ligaments and tendons, alteration of the articular surfaces, adhesion between them, and finally, ossification. He says, with great justice, that inflammation is essential to the production of this result, and that immobility, too much prolonged, greatly favors it.

Practically, this view is of much importance, as it shows the great value of antiphlogistic treatment, during the period of inflammation, and the absolute necessity of movements being made after this has subsided.

The necessity of resorting to passive movements, for the purpose of preventing the occurrence of anchylosis of the joints, is so apparent, and so much insisted on by surgical writers, it is scarcely requisite to insist upon it here. It is however, so frequently neglected or performed in such an imperfect or insufficient manner, or entrusted to the patient or his friends, when it should be carefully done by the surgeon himself, that it may be well to dwell upon it for a moment. The term "passive motion" signifies movements given to joints by a force foreign to the muscles of the member. Most commonly this force is applied by the hands of surgeons, and may vary in amount, from the slight pressure required in the most trifling cases, to an amount as great as he can well employ. "After the 20th day, Dessault and Ruyer took off the splints, and forced the fore-arm to movements of flexion and extension; and the success which followed this treatment, is a peremptory reason for its imitation."—*Malgaigne.*

Anchylosis after injuries, is to be guarded against, not only by "passive motion" but also by regulation of the position. After fractures or injuries of the elbow joints, this articulation should be kept flexed at about a right angle with the arm. "What is most important to be known in this case is, that long continued rest only leads to anchylosis when the member is extended. The fore-arm should be kept bent to a right angle." —*Malgaigne.*

"When the elbow cannot be prevented from becoming anchylosed, the joint should always be kept bent."—*Cooper's Dict.*

In these cases the joints must be kept bent angular, splints applied, and antiphlogistic treatment made use of.—*Erichson, p.* 251.

Not to dwell unnecessarily on a point not disputed that I know, let the opinion of Sir Astly Cooper in addition suffice: "The treatment consists in bending the arm. The best splint for it is one formed at right angles, the upper portion of which should be placed behind the upper arm and the lower portion under the fore-arm."—*On Dislocations and Fractures of the Joints, p.* 402.

TREATMENT OF THE RIGIDITY WHICH FOLLOWS INJURIES OF THE JOINTS.

While surgical writers and teachers are pretty unanimous in regard to the method of preventing anchylosis after sprains, dislocations, and fractures—the same unanimity does not by any means exist concerning its treatment after it has been allowed to take place. We have seen how great is the liability to stiffness and anchylosis with good care: without it, they are inevitable.

Ignorance of the practitioner, and want of docility on the part of the patient, are often causes, but more frequently the surgeon, guided by the authority of other times, trusts to inert and insufficient remedies. Fomentations, frictions with various kinds of oil, warm baths, mineral waters, etc., are the remedies often relied upon. Malgaigne has fully done justice to this kind of treatment. Speaking of it in connection with trusting to time and use of the part, he says, there is nothing more empirical and more dangerous than these modes of treatment. *The only remedy for a stiff joint is movement.*

"If a patient is abandoned to himself with a very slight degree of stiffness, it is simply to condemn him to incurability. I have already cited a sufficient number of cases of this kind; but that which I shall now give, will put the danger of such expectant treatment beyond all doubt." After giving a case, submitted to friction, warm bath, mineral water, which only leaves the member with anchylosis, he proceeds to lay down the rules for proceeding in such cases in the following words:

"*Consequently the surgeon must* (the dressing having been dispensed with) *move the joint, force the movements of those which are stiffened, and direct the patient to exercise it.* The following days the same treatment should be resorted to, until the joints shall have recovered their entire liberty."—*p.* 297.

This treatment would alarm some surgeons, and the pain would, unless anæsthetics were employed, scarcely be endured by many patients.

In reference to the pain attendant, and the symptoms which may be expected to follow, Malgaigne makes the following statement: "When we endeavor to move these articulations, it is not the muscles which constitute the principal obstacle, it is the ligaments; if we force the movement a little, pain is felt in the ligaments, tumefaction takes place around the joint, and all the symptoms following sprains result, and from the same cause —viz., stretching of the ligaments."—*p.* 136.

The treatise of Malgaigne, from which we have quoted, was published in 1847, and it is since that time that the practice he recommends has been generally adopted by the profession in various countries.

It had indeed long been known to the profession, that the movements of stiff joints had occasionally been restored by accidental violence.

A case had often been cited from Job A. Mekreen, of an old anchylosis of the elbow, which had resisted fomentations and cataplasms. He got a violent fall upon the fore-arm, and from that time the movements were re-established, and became thenceforth, from day to day, more extended and easy.—*Dict. de Medicine*, Art. Anchylosis.

Fabricius Hildanus relates a case of an anchylosis of the fingers and wrist, which was cured by a fall, which also fractured the fore-arm.

Bartholine mentions the case of a patient who had his arm dislocated; anchylosis followed, which was cured by a fall from a horse the following year.—*Velpeau, Operative Surgery, vol.* i, *p.* 478.

These, and other cases similar, were well calculated to direct the attention of the surgeons to the propriety of *breaking* up anchylosis, whether true or false, had they not been counterbalanced on the other hand by cases in which violence, too great and applied *mal apropos*, did great injury. The same remark is applicable to those cases of cure of anchylosis effected by so-called bone-setters. Boyer, Pellitan, and Marjolin have mentioned the cure of the Duchess of Luyanes, who had a stiff elbow, the result of an injury. There was false anchylosis.

"Several eminent surgeons were consulted, who prescribed the usual remedies without success; every time they attempted to move the joint, she cried out, and they were obliged to desist. At length she sent for a celebrated bone-setter, who had had great experience in such cases, who seized the fore-arm with one hand and the arm with the other, and straightened and bent it several times. At the end of forty-five days she was well."—*Marjolin.*

Boyer quotes from L. Verduc, the case of a young girl, from ten to twelve years old, who had the right knee anchylosed, the result of a wound between the patella and condyle. A physician and three surgeons considered the case incurable. Nevertheless, Verduc did not despair. After having fomented the knee with liquids, he seized the leg and thigh with his two hands and performed flexion and extension to as great a degree as he was able. Every day, morning and evening, flexion and extension were made with violence. In these extended movements, the sound produced by the friction between the tibia and fibula were heard. Frequently, after having performed these movements, it was necessary to leave the patient in a state of repose for seven or eight days, and as soon as she was better the flexion and extension were commenced. By this means the patient was cured.

Such was the state of science when, in 1840, a French surgeon, by the name of Louvrier, invented a machine for the breaking up of anchylosis. After having used it with full success in eighteen cases of false anchylosis, he was so emboldened as to apply it in some cases in the Paris Hospitals, which had been caused by white swelling, and on a patient affected with syphilis and scrofula.

Two of these patients, thus operated on, died from suppuration and laceration of the skin and muscles.

The subject was brought before the Academy of Medicine, who condemned it in a qualified manner. The report was read by Berard, at the sitting of the 27th of April, 1841, the conclusions of which were:—

1st. That the machine of Louvrier is followed by the instantaneous straightening of the member.

2d. That this straightening of the limb is ordinarily followed by no accident, either immediate or remote.

Notwithstanding these conclusions, the opinion of the Academy was unfavorable, and under its influence many surgeons, who at present are in favor of using suitable force, condemned not only this machine but also all attempts at forced movements. There were, however, not wanting those who thought that a method of treatment, which had according to the report been successful in eighteen cases of anchylosis, could not justly be condemned for having failed in two cases resulting from scrofula, syphilis, and white swelling, in which it ought never to have been used.

This opinion found expression in a work of the celebrated surgeon, Mayor, of Switzerland, published at Paris, in 1841, entitled, *On the Accelerated Treatment of Anchylosis*, in which this method was justly appreciated and favorably judged of, in its aplication to false anchylosis. Numerous cases were cited in it, of successful treatment by his method.

It was in this same year, 1841, that the work, *Dieffenbach on the Division of Tendons with Forced Rupture*, made its appearance. "He instituted the practice of violent rupture in addition to that of tenotomy and gradual extension, in order to combat those impediments which these had shown themselves unable to be subdued. It was now, that forced rupture, which the pathological anatomy so imperatively demands, was first legitimately received among the resources of the surgical art. We say legitimately, for prior to this time chance has been known to have produced cures by its agency."—*Frank*, *London Lancet*, *vol. for* 1853, *p*. 202.

"In cases of contractions of, and anchylosis of the knee-joint, Dieffenbach first performed the sub-cutaneous dissection of the flexor tendons, and of all contracted portions of the fascia, after which a towel having been firmly bound around the joint, the adhesions between the articular surfaces were ruptured by forcible flexion; thereafter, the joint was forcibly extended, and the apparatus of Stromeyer (that for gradual extension by a screw) was applied."

In 1850, Bonnet of Lyons, and Palasiano of Naples, published an account of a method which did not differ essentially from that of Dieffenbach.

We cite two of the cases of Mr. Meyor, as indicative of the extent to which he carried out his views in practice.

The first was of a lady, who had the knee anchylosed at a right angle, the result of an accident. Mr. Meyor seated her in a chair, put his left arm behind the knee, and when she was least expecting it gave the leg an impulse which bent it. It soon regained its movements.

The second, was an anchylosis of the knee in a straight position, which was also bent by violence and the movement restored.—*Bonnet, p.* 393.

Mr. Bonnet, in his work published in 1853, on the therapeutics of diseases of the articulations, gives the following direction for treating anchylosis of the knee: "The thigh should be brought to the edge of the bed, the surgeon places the left arm behind the upper part of the leg, and the right hand on the lower part of it in front, and gives it several pushes tending to bend it; a cracking is heard in the joint, and by degrees it gives way and bends. One movement of flexion is insufficient, it should be repeated five or six times by energetic movements produced by the hands of the surgeon and his aids."

Mr. Bonnet cites a large number of cases treated in this manner:—

1. A case treated by M. Palasiano, successful.
2. A case treated by M. Bouchacourt, also successful.
3. A case treated by himself, successfully.
4. This was a case of great difficulty, but the anchylosis gave way with a loud crackling sound, after great force had been used by the hands at five different times.
5. This case also required great force for five minutes.

6 and 7. Two other cases are given, treated with success in the same manner.

But the work of Bonnet is particularly valuable for my present purpose, from having a chapter devoted expressly to stiffness of the elbow.

To "the elbow, as to all the joints, we can, in case of stiffness, give artificial movements. For this purpose, the arm should be fixed, and the fore-arm alternately bent and extended. This generally requires one or two assistants, and in order to dispense with these, it is generally better to use a machine. This should consist, first, of a groove fixed upon a plank, into which the arm should be fixed; secondly, of two parallel shafts, between which the fore-arm is fixed by a sort of bracelet; thirdly, of the arc of a circle, graduated so as to measure the extent of the movements."

The cases cited of stiffness, removed by the use of this machine, are instructive, and I give some of the most unfavorable:

8. "A young man, aged sixteen years, fell upon the breast, and injured the elbow. I saw him five days afterwards, there was great pain, and the fore-arm could neither be flexed nor extended. I extended the fore-arm, flexed it, drew upon it strongly. The movements were quite restored in a few days."

9. "A young man, aged fifteen and a half years, had a dislocation of the elbow, which was reduced immediately, but inflammation followed and then a fibrous anchylosis remained. The joint was bent and extended by the machine, and this treatment was continued several weeks. Pretty free movements were restored."

In cases of anchylosis, resulting from old dislocations, Bonnet recommends breaking up the adhesions forcibly. "To execute these movements, I seize the contiguous extremities of the arm and fore-arm, and force them in opposite directions. After some efforts I feel the cracking, which proves the rupture has taken place." He adds that he has employed this method six times.

In cases of immobility, resulting from white swelling, cases the most unfavorable of all, Mr. Bonnet has found forced flexion and extension succeed.

10. "A young girl, aged nine years, had a disease of the elbow, without any appreciable cause. The joint became stiff.

It was treated by energetic movements of flexion and extension over a week, and the motion kept up by the machine in the intervening time. At the end of a month, the movements of flexion and extension could be performed to the extent of a quarter of a circle."—*Bonnet, p.* 551.

11. "Another young girl, of the same age, affected by scrofulous disease of the elbow, which was in a stage less advanced. The same treatment was used, which effected a perfect cure."—*p.* 552.

12. "In the case of a child, aged twelve years, affected with scrofulous disease of the elbow, suppuration occurred and an anchylosis almost perfect. Movements with the hands and the machine were employed, and at the end of four months, motion was in a great degree restored."—*p.* 553.

In all these, and similar cases, Mr. Bonnet adds, "It is unnecessary to say that graduated movements were insufficient, and, in order to succeed, strong tractions must be resorted to and extended, and energetic movements given to the fore-arm."

13. "A young man, of Montheis, eighteen years old, had an anchylosis of the elbow, of three years' standing, and of such a degree that the anterior face of the fore-arm was in contact with the arm. In December, 1849, after having ascertained, during insensibility from ether, that the anchylosis was complete, I divided the biceps which projected under the skin, and by alternate movements of flexion and extension succeeded in breaking the anchylosis, and extended it to an angle of ninety degrees with the humerus. Eight days afterwards, the patient returned home, taking a machine for movement with him."—*p.* 155.

16. "Mademoisselle Puy, aged fifteen years, was entrusted to my care for a white swelling of the elbow, the 27th June, 1852. The white swelling was far from being cured, and the anchylosis was perfect in the extended position.

"June the 30th, the patient being under the influence of ether, I broke up the adhesions with the greatest difficulty, but at length succeeded and brought the joint to a right angle. The rupture of the anchylosis was followed by acute inflammation, fever, and numerous abscesses formed around the joint.

I opened the abscesses with the knife at a white heat, and July 27th she was able to make use of the apparatus for movement.

"August 30th, the patient left the city, she having been able for a week to play the piano."—*p.* 155.

The authority of Bonnet as a writer on diseases of the joints, and the fact that he had five years previously condemned the practice of forced rupture, tended much to give currency to the practice. His method consisted in first flexing the member, then extending it, and retaining it by suitable dressing. The practice of first flexing, then extending the joint, instead of extending in the first instance, constituted the essential difference between the method of Dieffenbach and Bonnet, and that which had previously been adopted; a difference which it is important to bear in mind, as it constitutes the principal reason of the favorable results which have in recent times been attained. Among the list of those who contributed about this time to perfect the practice in these cases, we must not omit to cite Langenbeck, who used forced flexion and forced extension.

Nelaton who, in his *Elements of Surgical Pathology*, published in 1847, did not fully recommend this method, in 1853, before his class, treated a patient by the method of Bonnet.

It would lead me into endless citations, were I to quote all the authorities which might be cited in favor of the practice of forced flexion and forced extension in anchylosis. It may indeed be considered the established practice at the present time on the continent of Europe. I shall, therefore, conclude this part of my notes, by adding the practice of some of the leading surgeons of England.

Dr. Little published in 1853, (London,) a work on deformity, in which the forcible rupture of anchylosis is fully justified. Dr. Little says, with much truth :—

"The introduction of anæsthetics has rendered possible the general adoption of this practice." "The art of surgery has, however, recently received an invaluable addition to its means of usefulness by the discovery of the anæsthetic properties of chloroform and ether. By chloroformization, the two great obstacles to the employment of force to straighten a bent or contracted limb—namely, pain and voluntary muscular resistance—are removed. As soon as these impediments disappear, the hands of the single operator applied to the parts encounter the physical resistance only of the deformed parts, he can feel his way in the application of greater force; feels and perceives the resistance of parts successively overcome in an anatomical order; if greater rigidity still oppose, a few movements of the joint backwards and forwards prepare the way for a more extensive yielding. After straightening or bending the limb, as the case may have required, by this forcible procedure the part should be lightly secured in a retentive instrument, or on a common splint adjusted so as to maintain the position more favorable than that which obtained before the operation, though not in the position into which the hands of the surgeon may have brought it. For as soon as the effect of chloroform on the sensorial system disappears, the patient will arouse to the conviction of the violence which may have been employed; the part may be acutely painful, and incapable of sustaining the pressure of a tight bandage or ligature."

Mr. Ferguson who, in his work on Operative Surgery, was extremely severe upon all attempts to break up adhesions by force, we find now among the advocates of forcible extension and flexion. The following cases, treated by him only a few months since, will show his changed opinions and practice on this subject:—

"1. A case of old dislocation of the elbow-joint, was operated on by Mr. Ferguson, on the 13th of October, 1855. The parts were anchylosed, and out of place for four years; the patient suffering very much from a stiff elbow. Blisters and country air had been tried in vain, and, as a last resort, the young man wished the joint to be cut away or the limb amputated. Mr. Ferguson, as soon as he had had him placed fully under the effects of chloroform, proposed to break up all the old adhesions about the joint, which he succeeded in effecting by using considerable force, and thus restored him full power of using the limb.

"2. In a similar case, which had resisted the power of Mr. Lawrence to break up the attachments, he proposed to divide, by a sub-cutaneous section, the tendon of triceps; but the parts ultimately gave way. Mr. Lawrence said he had now tried it in several cases, more especially in children, and all his cases had turned out well; (this he also knew to be the experience of some other surgeons—amongst the rest, his friend M. Langenbeck, of Berlin;) the only precautions being that no active inflammation be present."—*London Lancet, for Feb.*, 1856.

Mr. Erichson, of the University College Hospital, London, author of a popular work on Surgery, seems also to have changed his views and practice in cases of anchylosis. For although, in his work, he does not condemn forcible extension, yet he seems to give the preference to slow rather than sudden movements; whereas, his practice at the present time is to use force freely. The following cases, from the *London Lancet*, recently treated by him, are extremely instructive:—

"The plan of treatment adopted by Mr. Erichson, is to place the patient fully under the effects of chloroform, so as to relax the muscles of the limb; then, as the lesser evil of the two, forcibly to straighten or extend the leg. In doing this, we noticed on more than one occasion loud snaps or cracks in the joint, not a little alarming at first, though without damage.

"Case 1. A young woman, aged twenty-two, who had been attacked, April, 1855, with acute rheumatism, and rheumatic contraction of the left knee, the latter bent at nearly a right angle and excessively painful, came under Mr. Erichson's care, who succeeded in straightening the limb, under chloroform.

"Case 2. A man, aged twenty-three, came under care in July, with contraction of the left knee, which was bent at right angles, and had been in this condition for eight months, the result of inflammation of the joint. There was no pain or uneasiness about it; very limited motion, not more than about two inches, when he attempted to move the foot. Mr. Erichson had him placed under the influence of chloroform, and then forcibly stretched the limb; loud crackling noises were heard, as if something was being crunched or torn through. There were no inflammatory symptoms subsequently; and he was able to leave the hospital in ten days, merely wearing starch bandages.

"Case 3. A woman, aged thirty; anchylosis nearly complete of the left knee for nine months, consequent on rheumatism; scarcely any motion in the joint; no pain or tenderness. The limb here, also, was straightened, or forcibly extended, under the influence of chloroform. The extension was attended with loud snapping or crackling of the old adhesions. Some severe inflammatory action followed, which, however, was easily subdued by evaporating lotions. This woman, also, had starch bandages applied, and left the hospital quite improved in health.

"Case 4. A woman, aged 42, whom we saw operated upon early last month. She complained of anchylosis of the knee, with bent or twisted condition of fore-arm and hand, all the result of rheumatic inflammation, contracted ten years since. Mr. Erichson straightened the limb, as usual, under chloroform; though, at first, he believed that he should have had recourse to division of the tendons. Loud crunching sounds were heard, as in the former cases. This has also done well.

"Case 5. This was an instance of anchylosis of the left knee, in a female aged thirty-two, of not less than sixteen years' standing, during the whole of which period the limb had not been put to the ground.

"The leg was considerably wasted, though nearly as long as the healthy limb; it was bent nearly at a right angle; the hamstring tendons were very tense. Mr. Erichson having placed the patient under chloroform, divided these tendons, and then forcibly straightened the limb, tearing down, apparently to us, several old adhesions, in and around the joint. No inflammatory action supervened; and this limb is now in a straight position, supported by starch and gum bandages.

"These cases may not claim any superiority over many others of a like kind by their originality, yet they are deserving of notice for their practical value."—*Lancet, August* 18*th*, 1855, *p.* 145.

Mr. Solly, Surgeon of St. Thomas' Hospital, also adopts the treatment of forcible flexion and extension in anchylosis. The following case, reported in the number of the *London Lancet*, for March, 1856, will sufficiently show the extent to which he carries it:—

"Thos. B——, aged 20, cheesemonger, was admitted under my care, 19th June, 1855. His left knee is perfectly fixed and immovable, the leg is bent upon the thigh, so as nearly to allow the toes to touch the ground. The foot and leg are not in a straight line with the thigh, but twisted outwards, so that the deformity is very considerable. The patella is adherent to the external condyle. There is no pain, tenderness, or swelling of the joint; but the limb is quite useless to him. The history is, that this condition of the joint followed an attack of acute inflammation, which was treated during a sojourn of twenty-one weeks in a Metropolitan hospital. His general health is good. In my first examination of this joint, I thought that the division of the hamstring muscles would materially assist me in straightening the limb, as they were in great tension; but when he was placed completely under the influence of chloroform, this disappeared, and true osseous anchylosis alone retained the leg in its false position. This bony anchylosis was, with some force, completely broken through, and the rending asunder of the bones was distinctly audible throughout the theatre with a loud crack, and the limb was straight. After his removal to bed, and before he had completely recovered his consciousness, the limb was firmly bound to a long back splint, with a foot piece, such as we use for a fractured patella.

"In thirty days, the patient left the Hospital quite well."

To what extent rupture of anchylosis is practiced in the United States, I am unable to say. Dr. Mott, as it is known, used it extensively in immobility of the lower jaw, and even employed the lever and screw figured by Pare, and the older surgeons for the purpose. Many of the American surgeons have adopted the much more severe operation of Barton.

I have been informed, that Dr. Gross, of Louisville, has used the practice of breaking them up with great success.

Dr. Brainard has used forced flexion and extension, in stiffness after fracture, dislocation, and sprain, for the last sixteen years; having found extension, by a graduated screw, insufficient, and as it cannot be accomplished while the patient is under the influence of chloroform, therefore too painful.

One of the cases treated by Dr. Brainard, was that of a young boy, about 10 years old, who had a fracture of the internal condyle of the humerus. False anchylosis resulted, the parents being unwilling to have the boy submitted to passive movements. After twelve weeks, however, finding the anchylosis was becoming more perfect, instead of being relieved by the use of frictions, fomentations, etc., forced flexion and extension was resorted to.

The force required was considerable, and the pain and swelling which followed, lasted for several days; but, by repeating the flexion, the movements of the member were very perfectly restored.

Another case of false anchylosis of the elbow, treated by Dr. Brainard, was one accompanied by an unnatural dislocation of the elbow, of five months' standing. The operation of breaking up the anchylosis and reducing it, was performed before the class of Rush Medical College, and a report of the case published in this *Journal* for 1847. The arm was in the straight position. The bending was effected by taking hold of the arm and fore-arm with the hands, and using the knee as a fulcrum. The result was extremely satisfactory, the elbow being bent and the movements restored to a great extent.

I have myself treated a case of this kind, of which I add the notes in conclusion of this part of the subject.

Patient, named James Hutchins, presented himself on the 1st of August, 1855, with false anchylosis of the right elbow-joint. The position of the arm nearly straight, and immovable, with some œdema about the joint injury of the ulnar nerve; with partial paralysis. According to the patient's statement, he had, two months previously, fallen from the hurricane deck of a steamboat, causing a dislocation of the bones of the fore-arm backwards, with fracture of the inner condyle, which was still evident. The dislocation was properly reduced at the time, but passive motion was neglected up to the time stated above.

*Treatment.*—August 1st, after administering the chloroform to insensibility, I broke up the adhesions at one operation, by forcibly flexing the arm over my knee, and again extending it. The amount of force required, was equal to my ability. The arm was placed at right angles, and supported with a sling, and the patient directed to return the next day.

August 2. Some slight inflammation had arisen with considerable pain; directed evaporating lotions, with a light bandage.

August 3d. The inflammatory action had subsided, and the arm was again flexed and extended with very little difficulty. I directed the patient to occupy the intervals of his visits at my office, in working the joint himself, also to use voluntary effort.

August 6th. Able to move the joint nearly one half, by voluntary effort. Again made forcible flexion and extension to the full extent, and directed as before.

August 10th. Capable of moving the joint, by voluntary effort, nearly two-thirds of the limits. I again used force, and moved the joint back and forth many times; directed friction with lard, in meantime to keep up as much movement as he was capable of producing.

August 14th. Patient began work at my house as a carpenter, and found but very little difficulty in using tools, excepting from weakness resulting from the protracted inactivity of the muscles. After four weeks from the first operation, he was earning full wages as a carpenter, and complained of no inability, excepting slight insensibility of the ulnar side of the forearm and hand, which had attended him from the first.

Those, in this country, who have considered Barton's operation of cutting down to the bone and sawing it through, as too severe to be justified, have generally regarded all cases of severe anchylosis as incurable.

Others have attached themselves exclusively to the notion of treatment by gradual extension by means of a screw. The reports of cases, made by Mutter, Chase, and others, are calculated to give an exaggerated idea of this method; on the one hand, it represents it as more successful than it really is, and on the other, as far less severe than it will be found in practice. Dr. Brainard, who published some cases treated successfully by it in 1844, has abandoned it, except as a means of producing passive movements to prevent anchylosis, or as a means of preventing its return after forced rupture.

This method is in fact the oldest known to surgery, and instruments for effecting it, are figured in Tubueivs and Guy de

Chauliac. But it has invariably fallen into disuse, and notwithstanding the recommendation of it by Chase and Mutter, it is now seldom employed by good surgeons, except for the treatment of club-foot, in which it is invaluable.

As erroneous opinions prevail on this subject, I will cite here the observations of Dr. Philip Frank, of Manchester, England, who was for many years assistant of Langenbeck, at Berlin, where, as well as in other cities of Germany, this method, with and without division of tendons, has been most thoroughly tried.

"Gradual extension, by means of pressure, or by the instrumentality of orthopedic apparatus, was the oldest method of treating contraction and anchylosis of the joints. This method has continued in practice to the present time; and many, who are either unable or unwilling to appreciate the advances of modern surgery, regard it as the only course commendable or judicious: we will, therefore, abstain from criticising this method, as practiced in former times, inasmuch as the results might be ascribed rather to the insufficiency of the apparatus in use, than to the method itself. Let us regard it in modern times, where the apparatus of Bonnet, Stromeyer and others, constructed on the most scientific principles, have come into use: still, how few satisfactory results have been obtained. It is not to be denied, that the method has been equal to the cure of simple contraction of the joints. Even cases of sprains, anchylosis with contractions at a small angle, have been successfully treated by gradual extension. Yet, even in these cases, simple as they were, the results were uncertain, the treatment tedious, inconvenient, and laborious. Excoriation and mortification supervening on those parts of the skin most exposed to the pressure of the machine; pains occasioned by continued tension; repeatedly exciting the muscles to unusual contractions; reflex action in different organs after a certain degree of extension had been arrived at: all these form a long series of evils, often enforcing protracted interruptions of the treatment, with loss of advantage already obtained, and sometimes total abandonment of the attempt."—*London Lancet, for* 1853, *p.* 202.

There is little doubt, that continued extension with a machine is more painful, and is attended with more danger of re-exciting inflammation, than forced movements with the hands, the patient being etherized.

It is moreover to be observed, that this gradual extension is not calculated to restore the movements of the member, but only to change its position: it has been used for extending, not for flexing. I have not been able to find on record any case in which a member, affected with anchylosis, was flexed, and the movements restored by the screw apparatus alone.

We shall not dwell upon the operation of Barton, as it has only been used for the femur and in the present condition, of our knowledge it is not likely to be again resorted to.

In conclusion, I think the following modes of practice and statements, may be considered as established by the best authorities of the profession and sound principles of surgery:—

1. Stiffness of the joints from injury should be treated and may be remedied by forced movements of the kind natural to the joint. They should be first used while the patient is under the influence of ether, and repeated as occasion may require.

2. Some imperfection of the movements, deformity of the joint, wasting of the muscles, and paralysis of the nerves, often remains after injuries of the elbow-joint, as the inevitable consequence of the injury.

